# Disruption of insect immunity using analogs of the pleiotropic insect peptide hormone *Neb*-colloostatin: a nanotech approach for pest control II

**DOI:** 10.1038/s41598-021-87878-5

**Published:** 2021-05-04

**Authors:** Patryk Nowicki, Mariola Kuczer, Grzegorz Schroeder, Elżbieta Czarniewska

**Affiliations:** 1 Department of Animal Physiology and Development, Institute of Experimental Biology, Faculty of Biology, Adam Mickiewicz University in Poznań, Uniwersytetu Poznańskiego Str. 6, 61-614 Poznań, Poland; 2 Faculty of Chemistry, University in Wrocław, F. Joliot-Curie Str. 14, 50-383 Wrocław, Poland; 3 Faculty of Chemistry, Adam Mickiewicz University in Poznań, Uniwersytetu Poznańskiego Str. 8, 61-614 Poznań, Poland; 4 Poznań, Poland

**Keywords:** Insect hormones, Nanoparticles

## Abstract

This work continues our studies on the pleiotropic activity of the insect peptide *Neb*-colloostatin in insects. In vivo immunological bioassays demonstrated that hemocytotoxic analogs of *Neb*-colloostatin injected into *Tenebrio molitor* significantly reduced the number of hemocytes in the hemolymph and impaired phagocytosis, nodulation and phenoloxidase activities in the insects. Among the analogs tested, [Ala^1^]-,[Val^1^]-, [Hyp^4^]- and [Ach^4^]-colloostatin were particularly potent in disrupting cellular immunity in larvae, pupae and adult insects. This result suggests that the most effective analogs showed increases in the bioactivity period in the hemolymph of insects when compared to *Neb*-colloostatin. Recently, we demonstrated that it is possible to introduce *Neb*-colloostatin through the cuticle of an insect into the hemolymph when the peptide is coupled with nanodiamonds. In this study, we showed that [Ala^1^]-, [Val^1^]-, [Hyp^4^]- and [Ach^4^]-colloostatin, when complexed with nanodiamonds, may also pass through the cuticle into the hemolymph and induce long-term impairments of immunity in *T. molitor* at all developmental stages. Studies on the tissue selectivity and effectiveness of *Neb*-colloostatin analogs and efficient methods for their introduction into insects may contribute to the development of eco-friendly pest control methods based on bioactive peptidomimetics.

## Introduction

Many insect species cause serious losses in agriculture, horticulture, forestry and food storage or transmit pathogens that cause disease in animals and humans; therefore, environmentally safe methods of reducing insect populations are intensively sought^1,2^. The use of nonselective insecticides is currently the most common method of pest control. Most often, insecticides disrupt the functioning of the insect nervous system by blocking receptors in neurons and voltage-gated sodium channels or inhibiting cholinesterase, making insects unable to breakdown acetylcholine in the synaptic cleft^3–6^. However, most insecticides have a negative effect not only on the target species but also on beneficial insects and on other animals and humans. The widespread use of nonselective insecticides results in a reduced susceptibility of pests to these toxins and, as a result, the need to increase the doses of the toxins, replace the insecticides to which pests have developed resistance with other insecticides, or use insecticide mixtures; these methods increase plant protection costs and cause the accumulation of these toxins in the environment and in food chains^7–9^.

Pest control methods alternative to nonselective insecticides include the use of natural enemies of pests, insect pathogens, bacterial toxins, genetically engineered pest-resistant crops or active molecules that are produced by plants and animals, including insects^1,10–16^. Insect hormones and peptides regulating physiological processes important for the life of an insect, i.e., homeostasis, metabolism, muscle contraction, immunity, reproduction and development, growth and behavior have a promising potential for the design of novel bioinsecticides^17–22^. Unfortunately, it is not possible to directly use natural peptides as bioinsecticides due to their poor pharmacokinetic properties and chemical instability. Therefore, it is necessary to design appropriately chemically modified analogs/peptidomimetics with increased (1) hydrophobicity that allows these molecules to overcome anatomical and physiological barriers to the target sites of an action, (2) resistance to peptidases contained in the hemolymph or intestinal fluid, (3) selectivity of organ action, and (4) agonistic or antagonistic activity in insects^19,23–34^. To date, many peptidomimetics that induce different biological activities in insects have been synthesized. Among them are peptidomimetics of insect peptides such as proctolin^26^, myosuppressins^20,27^, kinins^19,28^, pheromone biosynthesis-activating neuropeptides^29,30^, tachykinins^31^, sulphakinins^32^, allatostatins^33,34^ and trypsin modulating oostatic factor^17,18,21^.

One of the insect peptides that may be used in the future to reduce the number of pests is *Neb*-colloostatin (SIVPLGLPVPIGPIVVGPR), which is isolated from the ovaries of the flesh fly, *Neobellieria bullata*. This peptide was named colloostatin due to its structural similarity to invertebrate and vertebrate collagens and due to its oostatic activity^35^. The oostatic activity of *Neb*-colloostatin is not species-specific in insects because it has been shown to inhibit ovary function not only in *N. bullata*^35^ but also in *Tenebrio molitor*^36^ and *Hylobius abietis*^37^. The *Neb*-colloostatin-induced inhibition of oogenesis in insect ovaries results from the disruption of vitellogenin production in fat bodies as well as the inhibition of egg yolk deposition in oocytes through the modification of follicular epithelial patency, which leads to ovarian follicle atresia in females injected with this peptide^35–37^. Pico- and nanomolar doses of *Neb*-colloostatin induce hemocyte apoptosis in *T. molitor*^38^ and, as a consequence, significantly inhibit the cellular and humoral immune response at all developmental stages of the insect^39^. It was shown that nanodiamonds (NDs) can transfer this peptide through the cuticle to the hemolymph of the insect, where it exerts a proapoptotic effect on hemocytes, decreasing the number of hemocytes circulating in the hemolymph and impairing the innate immune response of *T. molitor*^39^. The structure–activity analysis of new *Neb*-colloostatin analogs in *T. molitor* hemocytes revealed hemocytotoxic analogs that were more potent than the native peptide^40^. However, the impact of these hemocytotoxic analogs on the immune response of insects has not yet been studied, and the increased hemocytotoxic activity resulting from these analogs suggests that they may strongly impair the innate immunity of insects; consequently, they may reduce the viability of insects more effectively than *Neb*-colloostatin can.

In this work, we examined the in vivo effect of proapoptotic analogs of *Neb*-colloostatin on the cellular and humoral immune responses (viability and number of circulating hemocytes, phagocytosis, nodule formation and phenoloxidase activity) of larvae, pupae and imagos of *T. molitor* to identify, among these compounds, the most effective analogs for reducing the immunity of the insects. We demonstrated that four *Neb*-colloostatin analogs are particularly active in decreasing the cellular and humoral immune response of the insects. Afterwards, we introduced these analogs through the cuticle into the hemocoel in complexes with NDs and showed that, compared to *Neb*-colloostatin, the analogs retained their specific hemocytotoxic activity in as similar manner but more effectively impaired the cellular and humoral immune responses of individuals of all developmental stages of the *T. molitor* beetle.

## Materials and methods

### Peptide synthesis, functionalization of NDs and preparation of the ND-peptide solution

The synthesis of *Neb*-colloostatin analogs ([Ac-Ser^1^]-, [Asp^1^]-, [Glu^1^]-, [Ala^1^]-, [Val^1^]-, [Hyp^4^]-, [Ach^4^]- and [Ile^4^]-colloostatin) was performed using a standard Fmoc procedure on Wang resins according to a previously described method^40^.

The noncovalent interactions between ND surfaces and peptides were used for the physical functionalization of NDs for *Neb*-colloostatin, [Ala^1^]-, [Val^1^]-, [Hyp^4^]- and [Ach^4^]-colloostatin according to a previously described method^39^. Peptides and ND-[Ala^1^]-, ND-[Val^1^]-, ND-[Hyp^4^]- and ND-[Ach^4^]-colloostatin were dissolved in a physiological saline of *Tenebrio* to yield stock solutions of 1 mg/ml. The prepared stock solutions were stored at –30 °C, and the working solutions were prepared in physiological saline just before use. The functionalized ND solutions were sonicated for 1 h before use to break up possible aggregates^39^.

### Insects

*Tenebrio molitor* used in the study were derived from a laboratory stock population maintained at the Department of Animal Physiology and Developmental Biology, Institute of Experimental Biology, Adam Mickiewicz University, Poznań, Poland. Insects were provided with ad libitum access to oat flakes and wheat flour with 5% w/v brewer’s yeast and supplemented with fresh carrot twice a week. The control and experimental groups (larvae, pupae and adults) were kept at the same population density in separate plastic boxes in a climate chamber at a constant temperature of 26 ± 0.5 ºC, with a relative humidity of 60 ± 5% and a photoperiod of 12 h of light and 12 h of darkness. All beetles used in our experiments were derived from parents that were less than 1 month old to avoid development disorders in the offspring. For each assay, the immunological studies were carried out on fifteen 120–140-mg larvae, 3-day-old pupae and 4-day-old adult beetles, with three biological replicates for each of the treatments.

### Immune response assays

After anesthesia with CO_2_, the *T. molitor* larvae, pupae and adults were split into control and experimental groups to investigate the influence of the hemocytotoxic analogs of *Neb*-colloostatin ([Ac-Ser^1^]-, [Asp^1^]-, [Glu^1^]-, [Ala^1^]-, [Val^1^]-, [Hyp^4^]-, [Ach^4^]- and [Ile^4^]-colloostatin) on the circulating hemocyte count (CHC), cellular immune response (phagocytosis and nodulation) and humoral immune response (phenoloxidase (PO) activity). The control insects were injected with 2 µl of physiological saline (274 mmol/l NaCl, 19 mmol/l KCl, and 9 mmol/l CaCl_2_), whereas the experimental insects were injected with 2 µl of *Neb*-colloostatin or an analog solution containing a dose of 2 ng of peptide per insect.

Then, the most immunologically active analogs ([Ala^1^]-, [Val^1^]-, [Hyp^4^]- and [Ach^4^]-colloostatin) were conjugated with ND and applied to the cuticle surfaces of larvae, pupae and adults to assess whether these conjugates impede phagocytosis, nodulation and phenoloxidase activity. After anesthesia, the control insects received 5 µl of saline, while the experimental insects were exposed to 5 µl of the ND-*Neb*-colloostatin, ND-[Ala^1^]-, [Val^1^]-, [Hyp^4^]- or [Ach^4^]-colloostatin solution containing a dose of approximately 4.15 µg ND and 0.85 µg peptide per insect.

### Testing the circulating hemocyte count

The number of hemocytes circulating in the hemolymph of larvae, pupae and adults of *T. molitor* was studied according to a previously described method^39^. After anesthesia, the saline-, *Neb*-colloostatin- and analog-treated insects were washed in 70% ethanol and rinsed in distilled water. To determine the CHCs, the collected hemolymph samples (2 µl) were diluted in 18 µl of physiological saline containing an anticoagulant buffer (4.5 mmol/l citric acid and 9 mmol/l sodium citrate, 5:1 v/v). Subsequently, the hemolymph samples were placed on a Bürker hemocytometer and analyzed under a light microscope. The CHC values were based on the number of hemocytes observed in 15 random squares in the diluted hemolymph samples and calculated by the following equation:

$${\text{CHC}} = {\text{a}} \times 10 \times 250$$

where CHC—the circulating hemocyte count; a—the average number of hemocytes in one square; 10—the dilution of the hemolymph sample; 250—the coefficient used to calculate the number of hemocytes in 1 µl of the sample.

### Phagocytosis

The phagocytic ability of the hemocytes isolated from the control and experimental groups of insects at various stages of development was studied in vivo with latex beads (Sigma-Aldrich L1030) according to previously described methods^39^. Latex beads suspended in physiological saline (1:500 v/v) were injected into beetles 2 h after peptide injection or 1 day after ND-*Neb*-colloostatin, ND-[Ala^1^]-, [Val^1^]-, [Hyp^4^]- or [Ach^4^]-colloostatin topical application. Hemolymph samples were collected 1 h after the injection of the latex bead solution and incubated for 30 min on coverslips coated with poly-L-lysine at room temperature in the dark. Subsequently, the hemocytes were washed with saline, fixed in 3.7% paraformaldehyde for 10 min and stained with a DAPI solution for 5 min in the dark to visualize the nuclei. The prepared hemocytes were used for phagocytosis analysis under a Nikon Eclipse TE 2000 U fluorescence microscope. Photos of the hemocytes were taken with a Nikon DS-1QM digital camera and analyzed with NIS Elements AR 3.10 software. The data are expressed as the percentage ratio of hemocytes with phagocytosed latex beads to the total number of hemocytes seen in a single image. In each biological replicate, the number of phagocytes was estimated from 5–7 random images.

### Nodulation

The ability of hemocytes to form nodules in insects that have been experimentally infected with bacteria was studied according to a previously described method^41^. Two hours after the injection of *Neb*-colloostatin or an analog solution or after the topical application of an ND-peptide solution (ND-*Neb*-colloostatin, ND-[Ala^1^]-, [Val^1^]-, [Hyp^4^]- or [Ach^4^]-colloostatin), beetles at various stages of development were anaesthetized again, washed in distilled water, disinfected and then injected with *Staphylococcus aureus* solution in physiological saline (1:500 v/v; 5 μl, formalin-fixed suspension of essentially nonviable *S. aureus*; Sigma R S2014 Saint Louis, Missouri, USA). Positive control insects were injected with only *S. aureus* solution in physiological saline (5 μl). Nodule formation was measured on the third day of the experiment. The beetles were dissected to expose the nodules on the dorsal side of the hemocoels. Each insect body was pinned to a SYLGARD R silicone-filled Petri dish, and the hemocoel was washed with physiological saline. Next, the fat body and alimentary canal were removed. Nodule formation was studied with an Olympus SZX 12 stereoscopic microscope (Olympus Co., Tokyo, Japan), and 3 images of each beetle were taken with an Olympus U-LH100HG digital camera (Olympus Co., Tokyo, Japan). The number of nodules on the dorsal side of the hemocoel was counted in all of the beetles.

### Humoral immune response

PO activity in *S. aureus*-infected *T. molitor* larvae, pupae and adults injected with *Neb*-colloostatin or an analog solution or exposed to an ND-peptide solution (ND-*Neb*-colloostatin, ND-[Ala^1^]-, [Val^1^]-, [Hyp^4^]- or [Ach^4^]-colloostatin) was measured on the third day of the experiment according to a modified method described previously^42,43^. In brief, 1 μl of hemolymph was placed on white filter paper (Whatman No. 52) soaked in a 10-mM sodium phosphate buffer containing 2 mg/ml DL-DOPA (Sigma Aldrich). Subsequently, the filter papers with the samples were incubated for 30 min at room temperature and air-dried. The samples were then scanned with a Canon Lide 110 scanner (600 dpi, 8 bits, gray scale). Images have been transformed so that the darker samples correspond to the highest PO activity. Images of the samples were analyzed with ImageJ (ver. 2), measuring the color intensity of the samples in their central 40 × 40 pixel area. The PO activity assessment was based on the mean pixel value of each image.

### Statistical analysis

Statistical tests were carriers out using GraphPad Prism 5 software (Department of Animal Physiology and Development, AMU license). We used the Shapiro–Wilk test to assay the statistical comparison of research variants and the normality of distribution. Statistical analyses were performed using a one-way ANOVA and Tukey’s post hoc test and a Student’s *t*-test. All data were considered statistically significant at a *p* value < 0.05.

## Results

### CHC assay

**Figure 1:**
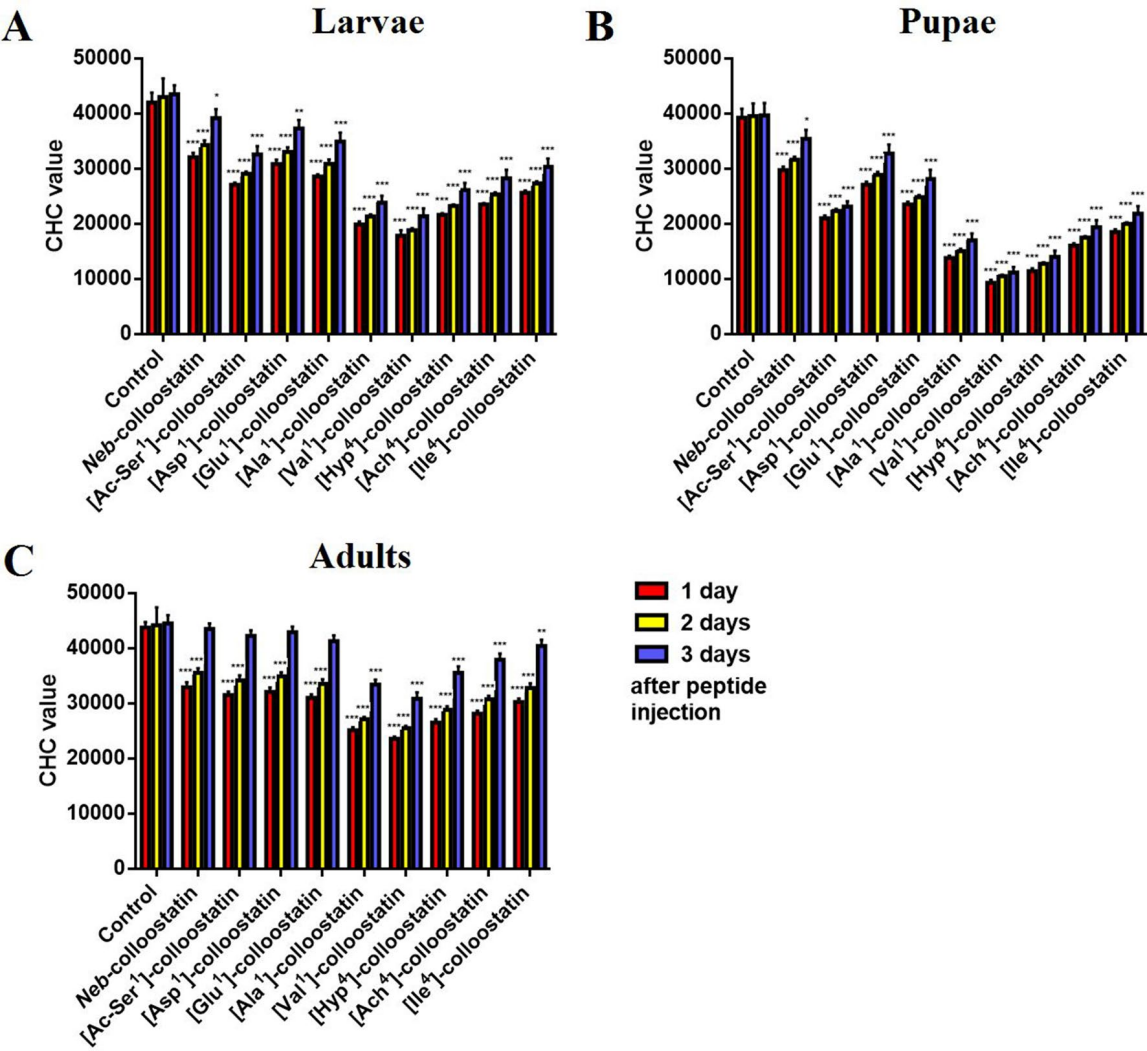
The circulating hemocyte count in *T. molitor* larvae (**A**), pupae (**B**) and adults (**C**) following an injection of *Neb*-colloostatin or one of its analogs at doses of 2 ng of peptide per insect. The values shown are means ± S.D. The results that are significantly different from those of the control group are indicated: *p* < 0.5 (*), *p* < 0.05 (**), and *p* < 0.005 (***).

The impacts of *Neb*-colloostatin and its analogs on the number of hemocytes in the hemolymph of *T. molitor* larvae, pupae and adults is shown in Fig. 1. In larvae and pupae, all tested peptides caused a significant decrease in the CHC value throughout the whole experiment (Fig. 1A,B). In the adult insects injected with *Neb*-, [Ac-Ser^1^]-, [Asp^1^]- and [Glu^1^]-colloostatin, a significant reduction in the CHC value was observed on the first and second days of the study, whereas in adults injected with [Ala^1^]-, [Val^1^]-, [Hyp^4^]-, [Ach^4^]- and [Ile^4^]-colloostatin, a significant decrease in the CHC value was observed over the course of the whole experiment (Fig. 1C). Among the tested peptides, four of the most active analogs were identified; two of the active analogs were modified at position 1 of the peptide chain ([Ala^1^]-, [Val^1^]-colloostatin), while the other two were modified at position 4 ([Hyp^4^]-, [Ach^4^]-colloostatin). These compounds were the most potent in reducing CHC values in all developmental stages of the studied insect. However, the hemocytes of pupae were more sensitive to these analogs than the hemocytes of larvae, and the hemocytes of adult insects were the least sensitive (Fig. 1A–C).

### Cellular immune response

**Figure 2:**
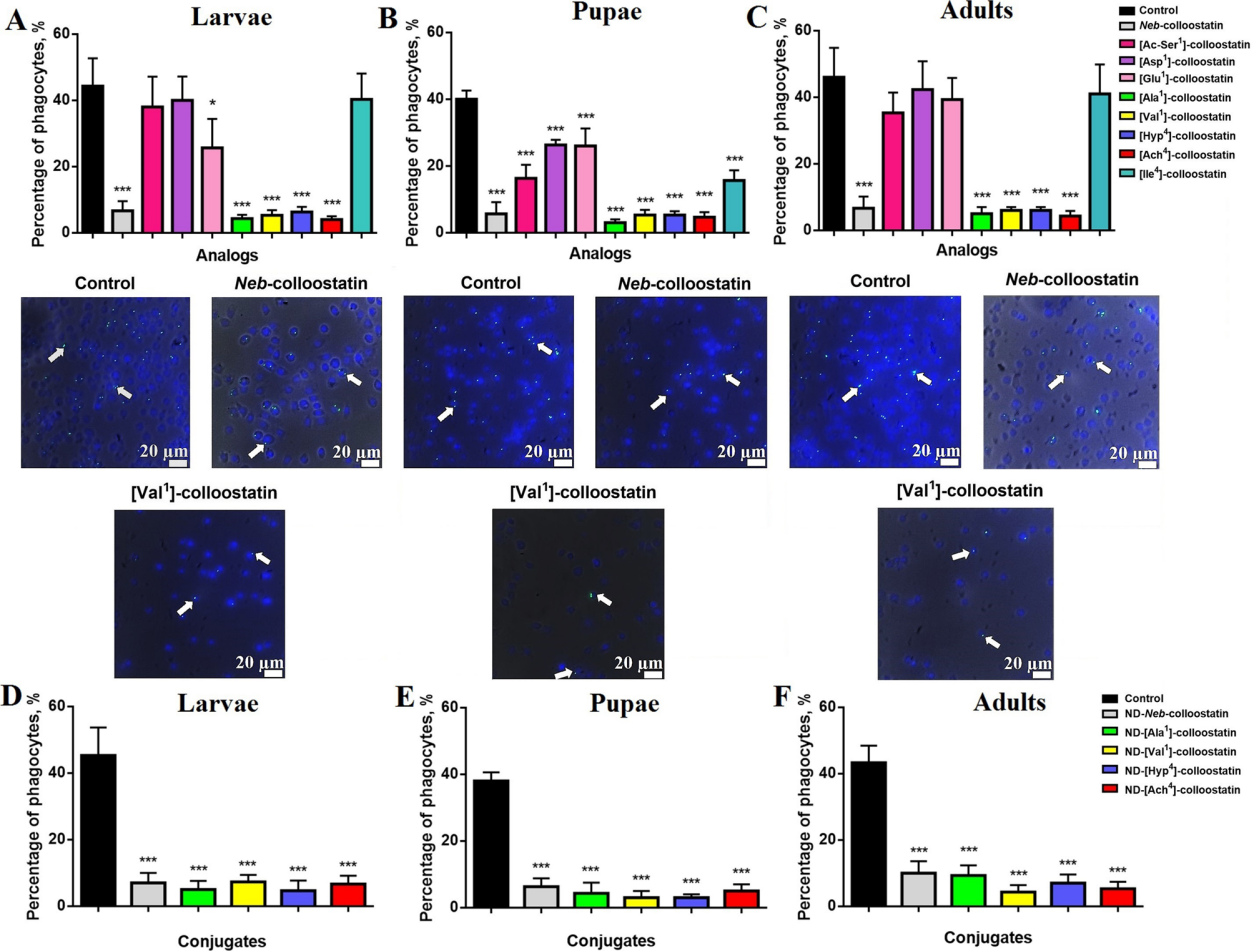
Phagocytosis in *T. molitor* larvae (**A**,**D**), pupae (**B**,**E**) and adults (**C**,**F**) following injections of *Neb*-colloostatin or its analogs at doses of 2 ng of peptide per insect (**A**–**C**) and following topical applications of ND-*Neb*-colloostatin or conjugates of NDs and the most potent analogs at doses of approximately 4.15 µg of NDs and 0.85 µg of peptide per insect (**D–F**). The values shown are means ± S.D. The results significantly different from those of the control group are indicated: *p* < 0.5 (*), *p* < 0.005 (***). Representative fluorescence microscopy images showing the phagocytosis of fluorescent latex beads by hemocytes of larvae, pupae and adult insects injected with saline (control), *Neb*-colloostatin and [Val^1^]-colloostatin. Arrows show phagocytes with fluorescent latex beads (green). Nuclei of hemocytes where stained with DAPI (blue).

The phagocytic activities of *Neb*-colloostatin, its analogs and ND-peptide conjugates (applied at a dose of 2 ng of peptide per insect or approximately 4.15 µg of NDs and 0.85 µg of peptide per insect, respectively) in all studied developmental stages of *T. molitor* are shown in Fig. 2. The analogs [Ac-Ser^1^]-, [Asp^1^]- and [Ile^4^]-colloostatin did not significantly impair the phagocytosis of latex beads when injected into larvae (Fig. 2A). The analogs [Ac-Ser^1^]-, [Asp^1^]-, [Glu^1^]- and [Ile^4^]-colloostatin, when injected into adult insects, also did not change the phagocytic activity of the hemocytes (Fig. 2C). The analogs [Ac-Ser^1^]-, [Asp^1^]-, [Glu^1^]- and [Ile^4^]-colloostatin, when injected into pupae, significantly reduced the ability of the hemocytes to phagocytose the latex beads; however, these analogs were less active in decreasing the phagocytosis of latex beads than *Neb*-colloostatin was (Fig. 2B). The largest reduction of phagocytosis of the latex beads, among the analogs tested, was demonstrated by [Ala^1^]-, [Val^1^]-, [Hyp^4^]-, [Ach^4^]-colloostatin in all developmental stages of the insects (Fig. 2A–C). The phagocytic activities of these analogs were similar to that of *Neb*-colloostatin. After the topical application of the most active peptides ([Ala^1^]-, [Val^1^]-, [Hyp^4^]-, [Ach^4^]-colloostatin) in complexes with NDs, we observed a strong reduction in the phagocytic activities of the hemocytes in larvae, pupae and adult insects (Fig. 2D–F). The decrease in the phagocytosis of latex beads caused by these complexes was similar to the reduction in phagocytosis observed after the topical application of ND-*Neb*-colloostatin.

**Figure 3:**
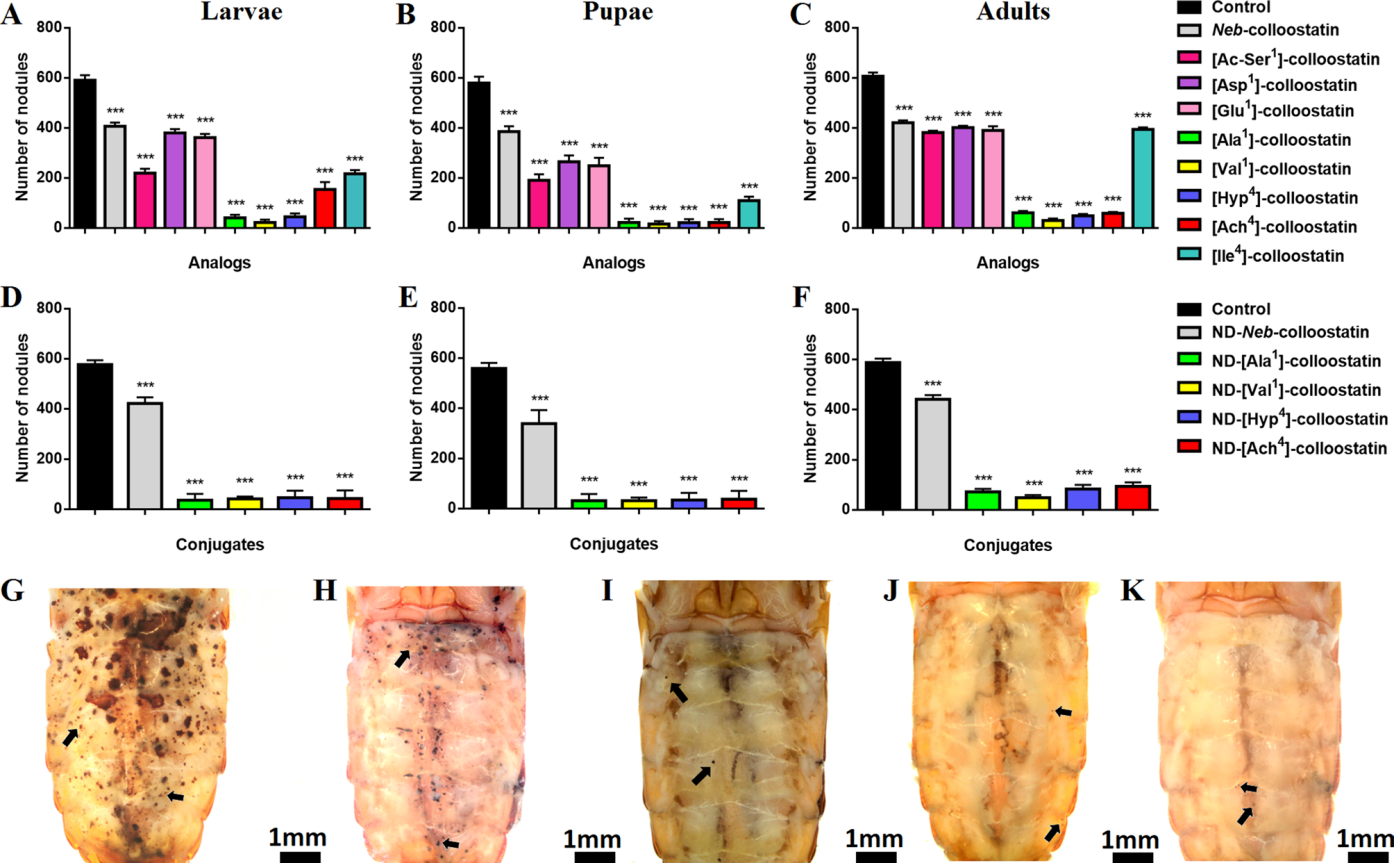
Nodule formation in *T. molitor* larvae (**A**,**D**), pupae (**B**,**E**) and adults (**C**,**F**) following injections of *Neb*-colloostatin or one of its analog at doses of 2 ng of peptide per insect (**A**–**C**) and following topical applications of ND-*Neb*-colloostatin or conjugates of NDs and the most potent analogs at doses of approximately 4.15 µg of NDs and 0.85 µg of peptide per insect (**D–F**). Representative photos are provided of the hemocoels of *T. molitor* adults taken 3 days after the injection of physiological saline (**G**), [Ala^1^]-colloostatin (**H**), [Val^1^]-colloostatin (**I**), [Hyp^4^]-colloostatin (**J**) or [Ach^4^]-colloostatin (**K**). Nodule formation was induced by injection of 5 *μ*L of *Staphylococcus aureus* in suspension. The black arrows show some examples of nodules. The values shown are means ± S.D. The results significantly different from those of the control group are indicated: *p* < 0.005 (***).

The long-term immunological study showed that the injection of peptides (in a dose of 2 ng of peptide per insect) and the topical application of ND-peptide conjugates (at a dose of approximately 4.15 µg of NDs and 0.85 µg of peptide per insect) caused a significant reduction in the number of nodules formed in the hemocoels of experimentally infected insects at all stages of development in comparison to the bacteria-challenged control larvae, pupae and adults (Fig. 3). We demonstrated that [Asp^1^]- and [Glu^1^]-colloostatin in larvae and [Asp^1^]- and [Ile^4^]-colloostatin in adult insects exhibited *Neb*-colloostatin-like activity (Fig. 3A,C). The second group of nodulation inhibitors with significantly increased activity compared to the native peptide included [Ac-Ser^1^]- and [Ile^4^]-colloostatin in larvae, [Ac-Ser^1^]-, [Asp^1^]- and [Glu^1^]-colloostatin in pupae and [Ac-Ser^1^]- and [Glu^1^]-colloostatin in adult insects (Fig. 3A–C). The third group of nodulation inhibitors that were extremely active included [Ala^1^]-, [Val^1^]-, [Hyp^4^]-, and [Ach^4^]-colloostatin in larvae and adult insect and [Ala^1^]-, [Val^1^]-, [Hyp^4^]-, [Ach^4^]- and [Ile^4^]-colloostatin in pupae (Fig. 3A–C).

The most active analogs, i.e., [Ala^1^]-, [Val^1^]-, [Hyp^4^]- and [Ach^4^]-colloostatin, when complexed with NDs and topically applied to the cuticle of larvae, pupae and adult insects, altered the number of nodules formed in the hemocoels of the bacteria-challenged insects at all stages of development in comparison to the experimental infected controls. The inhibition of nodule formation caused by these complexes in all studied developmental stages of *T. molitor* was greater than the inhibition of nodule formation caused by the topical application of ND-*Neb*-colloostatin.

### Phenoloxidase activity assay

**Figure 4:**
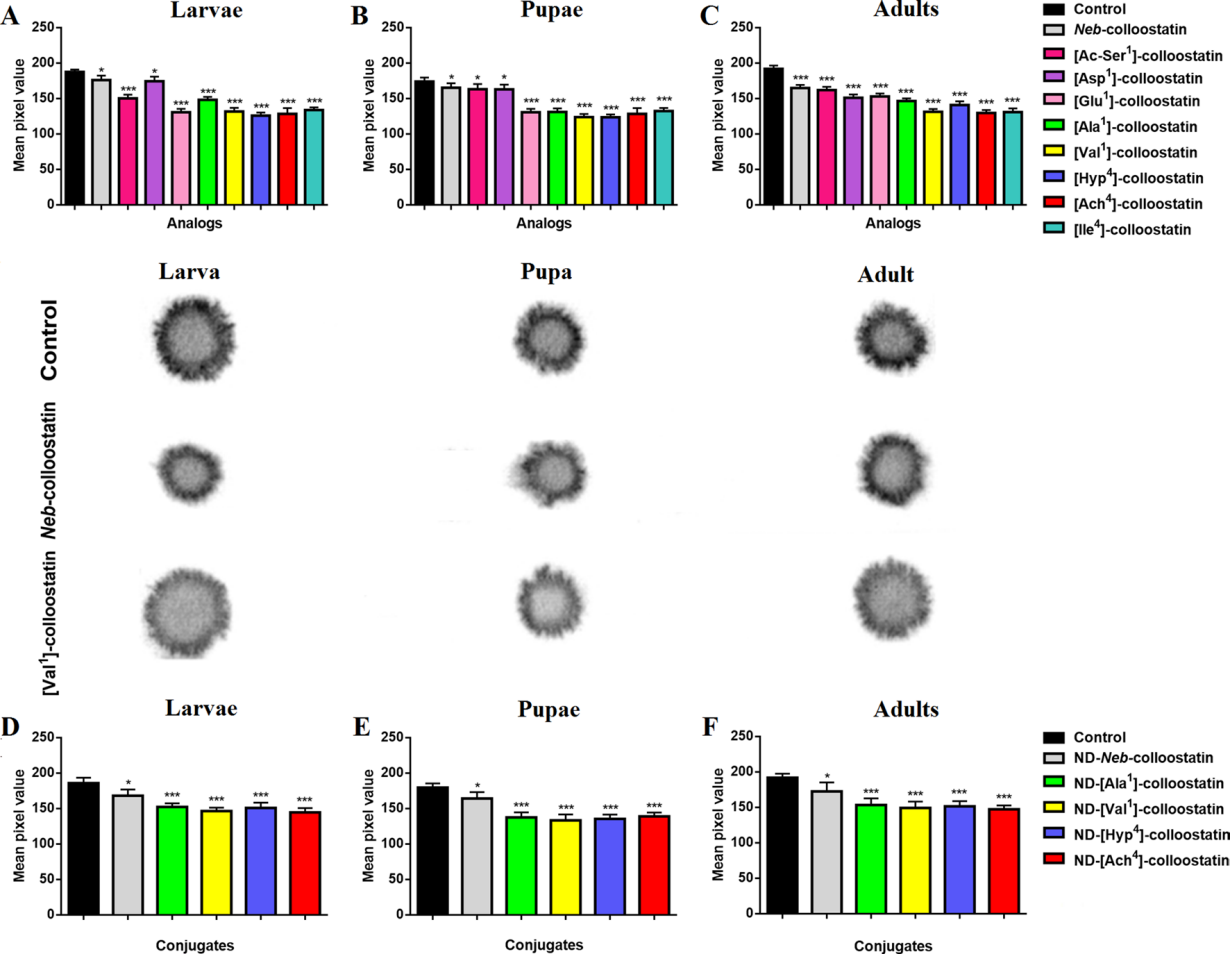
Phenoloxidase activity in bacteria-infected *T. molitor* larvae (**A**,**D**), pupae (**B**,**E**) and adults (**C**,**F**) following injections of *Neb*-colloostatin or one of its analog at doses of 2 ng of peptide per insect (**A**–**C**) and following topical applications of ND-*Neb*-colloostatin or conjugates of NDs and the most potent analogs at doses of approximately 4.15 µg of NDs and 0.85 µg of peptide per insect (**D**–**F**). The values shown are means ± S.D. The results significantly different from those of the control group are indicated: *p* < 0.005 (***). Examples of melanized spots produced by 1-µl aliquots of hemolymph taken from saline-, *Neb*-colloostatin- or [Val^1^]-colloostatin-injected larva, pupa and adult of *T*. *molitor.*

The PO activity in the hemolymph of the peptide-injected (at doses of 2 ng of peptide per insect), ND-peptide-exposed (at doses of approximately 4.15 µg of NDs and 0.85 µg of peptide per insect) and bacteria-challenged insects at all stages of development was compared to the PO activity of the saline-treated and bacteria-challenged controls (Fig. 4). Significant changes were observed in the PO activity in the hemolymph of larvae, pupae and adult insects resulting from injections of all tested peptides. We demonstrated *Neb*-colloostatin-like PO activity in larvae injected with [Asp^1^]-colloostatin, pupae injected with [Ac-Ser^1^]- or [Asp^1^]-colloostatin and adult insects injected with [Ac-Ser^1^]- and [Glu^1^]-colloostatin. Among the analogs that decreased PO activity more potently than *Neb*-colloostatin, we identified [Ac-Ser^1^]-, [Glu^1^]-, [Ala^1^]-, [Val^1^]-, [Hyp^4^]-, [Ach^4^]- and [Ile^4^]-colloostatin for larvae, [Glu^1^]-, [Ala^1^]-, [Val^1^]-, [Hyp^4^]-, [Ach^4^]- and [Ile^4^]-colloostatin for pupae and [Asp^1^]-, [Ala^1^]-, [Val^1^]-, [Hyp^4^]-, [Ach^4^]- and [Ile^4^]-colloostatin for adult insects. The most potent inhibitors of PO activity in hemolymph were [Glu^1^]-, [Val^1^]-, [Hyp^4^]-, [Ach^4^]- and [Ile^4^]-colloostatin when injected into larvae, [Val^1^]-, [Hyp^4^]-, and [Ach^4^]-colloostatin when injected into pupae and [Val^1^]-, [Hyp^4^]-, [Ach^4^]- and [Ile^4^]-colloostatin when injected into adult insects.

We compared the PO activity of ND-*Neb*-colloostatin, ND-[Ala^1^]-, ND-[Val^1^]-, ND-[Hyp^4^]- and ND-[Ach^4^]-colloostatin-treated and subsequently bacterially challenged larvae, pupae and adult insects in comparison to the PO activity of the controls. We demonstrated significant changes in PO activity in the hemolymph of insects at all developmental stages following topical applications of all ND-peptide conjugates in comparison to the PO activity of the control larvae, pupae and adult insects (Fig. 4D–F). All the most potent analogs conjugated with NDs caused greater reductions in PO activity than ND-*Neb*-colloostatin did.

## Discussion

In insects, the goal of the immune response is to quickly and efficiently remove pathogens or abiotic particles from the hemolymph^44^. Cellular immunity is tightly integrated with humoral immunity through the activity of the hemocytes circulating in the hemolymph of insects. The hemocytes phagocytose pathogens and other foreign bodies in response to infection and/or isolate the pathogens from other host cells through nodule formation or encapsulation. Phagocytosis is initiated by pattern-recognition receptors on the surface of hemocytes that recognize and bind to complementary molecules or their fragments that are present in an abiotic particle, pathogen or apoptotic body. During the immune recognition of pathogens by hemocytes, the hemocytes recognize complementary chemical signals from the pathogens, such as fungal-specific β-1,3 glucans, bacterial peptidoglycans and lipopolysaccharides, and the nucleic acids of bacteria and viruses^44^. The engulfment of a pathogen or abiotic particle by a phagocyte depends on the local polymerization of actin and the formation of F-actin microfilaments at the remodeling site of a fragment of the cell membrane, which then surrounds the absorbed object and forms a phagosome^45^. At the next stage of phagocytosis, pathogens are killed by reactive oxygen species (ROS), and the enzymatic degradation of pathogens takes place in phagosomes with the participation of enzymes such as V-ATPase, DNase and cathepsin^46^. It has been suggested that the hemocytes that effectively remove pathogens from the insect's hemolymph are plasmocytes^47^. However, our previous studies indicate that, in *T. molitor,* the ability to phagocytose latex beads is demonstrated not only by plasmatocytes but also by granulocytes and even prohemocytes (unpublished results).

Regarding nodule formation, it involves a large number of hemocytes in the isolation and inactivation of the invading pathogens^44^. Granulocytes are responsible for the immobilization of pathogens in the hemolymph and the chemotaxis of plasmatocytes to the site of infection. Granulocytes release the content of their granules into the insect's hemolymph, and together with plasmatocytes, they form a multilayered nodule that isolates pathogens from other host cells^48^. During nodule formation, remodeling of the hemocytes in the cytoskeleton occurs because the hemocytes that were previously freely circulating in the insect’s hemolymph must become adhesive cells at the site of infection. Hemocytes are also responsible for the synthesis and release of prophenyloxidase into the hemolymph of the insect, which, after activation in the hemolymph by serine proteases, is involved not only in the recognition, opsonization and killing of pathogens but also in the coagulation of the hemolymph, wound healing, and sealing of nodules and capsules that isolate pathogens from other cells of the insect^44,47^. The ability of insects to maintain an appropriate number of hemocytes in the hemolymph is therefore of key importance for the resistance of insects to pathogens and thus for their viability and survival.

As shown in Fig. 1, all tested analogs of *Neb*-colloostatin caused significant decreases in the number of hemocytes circulating in the hemolymph of *T. molitor* larvae, pupae and adults compared to the number of hemocytes observed in the control groups. The findings of this study showed that the analogs [Ala^1^]-, [Val^1^]-, [Hyp^4^]- and [Ach^4^]-colloostatin were the most effective in reducing the number of hemocytes circulating in the hemolymph of larvae, pupae and adult insects; the second group includes [Ac-Ser^1^]-, [Asp^1^]-, [Glu^1^]-, and [Ile^4^]-colloostatin, which also significantly decreased the number of hemocytes in the hemolymph of insects at all developmental stages but were less effective than the compounds belonging to the first group of peptides. The differences in the reduction of the hemocyte's number correlate with the proapoptotic activities of the analogs tested, specifically the proapoptotic activities of [Ala^1^]-, [Val^1^]-, [Hyp^4^]- and [Ach^4^]-colloostatin were higher than that of *Neb*-colloostatin by 75%, 129%, 113% and 85%, respectively, while the proapoptotic activities of [Ac-Ser^1^]-, [Asp^1^]-, [Glu^1^]-, and [Ile^4^]-colloostatin exceeded the *Neb*-colloostatin activity by 38%, 28%, 30% and 37%, respectively^40^. It is known that under physiological conditions, pathogen-induced macrophages and neutrophils produce and release large amounts of reactive oxygen species (ROS) that destroy intruders^44,49^. Despite the essential role of mitochondrial ROS in the response of immune cells to invading pathogens, mitochondrial ROS may also permeabilize mitochondrial membranes when overproduced and may consequently induce apoptosis of cells or cause organ failure^50–52^. We showed that *Neb*-colloostatin, when applied at picomolar and nanomolar doses, induced degenerative changes in mitochondria caused by disruption of the mitochondrial membrane potential, activated caspases in the cytoplasm and caused changes in the chromatin and F-actin cytoskeleton of hemocytes^38^. The doses of *Neb*-colloostatin and its analogs used so far in our investigations presumably promote significant increases in ROS formation in mitochondria, which in turn may lead to the permeabilization of the mitochondrial membrane and, finally, to hemocyte apoptosis. We presume that the analogs studied in this work may produce even greater amounts of ROS in hemocytes than *Neb*-colloostatin does and therefore, have higher proapoptotic activities and are more effective in reducing the number of hemocytes circulating in the hemolymph of larvae, pupae and adult insects. It is also possible that the *Neb*-colloostatin doses used so far in our studies significantly exceed the physiological range of doses of this peptide in the hemolymph of the studied insect^36,38,39^. If this hypothesis turns out to be true, it would be necessary to verify the opinion of *Neb*-colloostatin as an immunosuppressive peptide and consider the possibility that this peptide, when naturally produced at physiological doses in insect ovaries, may have an immunostimulatory effect on insects. However, further detailed research is needed to clarify this issue. We also showed in this study that following a significant decrease in the number of circulating hemocytes in the hemolymph of larvae, pupae, and adult insects injected with *Neb*-colloostatin analogs, the number of these cells in the hemolymph of insects at all developmental stages gradually increased in the time tested, but this increase did not compensate for the number of hemocytes previously lost by apoptosis. However, the exception to this rule are adult insects injected with [Ac-Ser^1^]-, [Asp^1^]-, [Glu^1^]-colloostatin, in which the number of hemocytes in the hemolymph reached the control value on the third day of the experiment (Fig. 1). The rate of increase in a total hemocyte population in the hemolymph was dependent on the type of chemical modification of the *Neb*-colloostatin molecule and on the developmental stage of the insect (Fig. 1). We suggest that chemically modifying the *Neb*-colloostatin molecule by introducing Ac-Ser, Asp, Glu, Ala or Val at the 1st position of the peptide amino acid chain or Hyp, Ach or Ile in the 4th position may impede access of the proteases contained in the hemolymph to the peptide bonds in the molecules of these analogs, and thus, the half-life of these analogs in the hemolymph of the insect may be extended. The results of the CHC study show that among the tested analogs, [Ala1]-, [Val1]-, [Hyp4]- and [Ach4]-colloostatin were the most effective in lowering the number of hemocytes circulating in the hemolymph and the cellular and humoral immune responses in *T. molitor*, which may suggest that these compounds are the more stable in the hemolymph of the insect. The gradual increase in the number of hemocytes circulating in the hemolymph of larvae, pupae and adult insects observed in the course of the study may not only be the result of differences in the half-life of the peptides in the insect hemolymph but may also indicate a gradual release of tissue-settled hemocytes as well as new populations of these cells from the hematopoietic organs. Restoration of the number of hemocytes in insect hemolymph was also found in *Bombyx mori* larvae after *Escherichia coli* injections^53^. Recently, we showed a similar effect in *T. molitor* individuals injected with either a solution of another immunomodulatory insect peptide, alloferon, or one of its analogs. We demonstrated that one day after the injection of these peptides into adult insects, apoptosis of hemocytes was observed, but later, a new pool of healthy hemocytes appeared in the hemolymph of the insect^41^. These findings indicated that hemocytes could have been released from the hematopoietic organs of the insects and that tissue-settled hemocytes could also be released into the hemolymph^41^. In the current study, we also demonstrated that the rate of restoration of the pool of hemocytes circulating in the hemolymph depends on the developmental stage of the insect. The increase in the number of hemocytes in the hemolymph was slowest in pupae, faster in larvae and fastest in adult insects (Fig. 1). It was also showed in a study of the effectiveness of JH analogs to interfere with the normal course of insect metamorphosis that the developmental stage of *T. molitor* and the house fly, *Musca domestica*, most sensitive to these analogs was the pupa, and in the bug *Oncopeltus fasciatus,* the last stage of the nymph was observed to be most sensitive^54^. This is because, during metamorphosis, the structure of an insect's body changes dramatically at the pupal stage owing to the simultaneous processes of histolysis and histogenesis; therefore, regeneration of the pool of lost hemocytes in pupae is probably more difficult than in larvae and adult insects^48^.

As shown in Figs. 2, 3, 4, the significant reductions observed in the number of hemocytes and the slowing of the hemocyte count recovery in the hemolymph at all stages of *T. molitor* development caused by the studied *Neb*-colloostatin analogs have serious consequences for the functioning of the immune systems of insects, expressed as a marked impairment of the cellular and humoral immune responses of the insects. We recently demonstrated that *Neb*-colloostatin significantly reduced latex bead phagocytosis, nodule formation and phenoloxidase activation in experimentally infected larvae, pupae and adults of *T. molitor* resulting from a pronounced decrease in the number of hemocytes in the hemolymph of the insects^39^. Therefore, it is not surprising that when larvae, pupae, and adult insects were treated with analogs that are more proapoptotic than *Neb*-colloostatin, reductions in the cellular and humoral immune responses of the insects were observed. Moreover, the study of the influence of *Neb*-colloostatin analogs on short- and long-term cellular immune responses in larvae, pupae and adult insects showed that [Ala^1^]-, [Val^1^]-, [Hyp^4^]- and [Ach^4^]-colloostatin were the most effective analogs in reducing not only the number of hemocytes in the hemolymph but also latex bead phagocytosis and nodule formation at all stages of *T. molitor* development.

Due to the multidirectional activity of PO in insects, disruption of the activity of this enzyme not only impairs the killing of pathogens by inhibiting the synthesis of ROS and quinones but also reduces the synthesis of melanins that seal the structure of nodules^48,55,56^. The release of prophenoloxidase into an insect's hemolymph is mediated by eicosanoids, which induce the lysis of hemocytes synthesizing this proenzyme^57^. However, during hemocyte apoptosis, cell lysis does not take place, but the dying cells shrink and form apoptotic bodies; therefore, it can be assumed that hemocytes that have started apoptosis as a result of the action of all tested *Neb*-colloostatin analogs are unable to phagocytose latex beads, form nodules around bacteria, or synthesize or release prophenoloxidase into the hemolymph of *T. molitor* larvae, pupae and adults that were experimentally infected with bacteria. On the other hand, a long-term effect of the hemocytotoxic action of the tested analogs was a gradual increase in the number of hemocytes in the hemolymph of larvae, pupae and adults of *T. molitor* (Fig. 1) and presumably for this reason, the efficacy of these analogs in inhibiting the humoral immune response was lower and less varied (Fig. 4) than was their effectiveness in reducing the cellular immune response in the bacterially infected insects (Fig. 3). Taken together, the results of the immunological bioassays seem to support our earlier hypothesis^40^ that the presence of hydrophilic Ser at position 1 of the *Neb*-colloostatin amino acid chain is not important for its biological activity. Additionally, this means that not only the electric charge but also the structure of the side chain of the *N*-terminal amino acid residue is important for the immunological activity of *Neb*-colloostatin.

In experimental entomology, the method of injecting peptide solutions directly into the hemolymph of an insect is widely used; this method avoids the proteolytic degradation of peptides in the insect's digestive tract. Thanks to this method, it is possible to study the relationship between the chemical structure and the physiological activity of a tested peptide. This scientific approach allows the identification of the organ specificity of a peptide and its derivatives as well as the identification of the core sequence of a peptide molecule responsible for the binding of the peptide to its receptor and thus responsible for its specific physiological activity in the tissues and organs of an insect^24,28,36,38,41^. In turn, understanding the peptide core sequence allows for appropriate chemical modifications of the peptide molecule to prevent rapid degradation of the obtained analogs and peptidomimetics by peptidases contained in the hemolymph, tissues and intestine of pest insects^19,23,28,35,37^. The use of peptide derivatives with increased activity relative to native peptides for pest control is an accepted idea and is used to a limited extent^17–34^. However, the future practical use of peptides or their protease-resistant analogs/peptidomimetics to effectively reduce the viability, reproduction and development of insect pests requires the development of alternative methods for introducing these molecules into an insect's body that are as effective as injecting peptides into the hemolymph of an insect. In vitro studies on the passage of chemically modified insect peptides through the cuticle model isolated from *Heliothis virescens*, *Periplaneta americana* and *Heliothis peltigera* have already been undertaken and have demonstrated that amphiphilic analogs of insect pyrokinin neuropeptides and pheromone biosynthesis-activating neuropeptides showed enhanced abilities to transmigrate the cuticles of insects in physiologically significant quantities without losing their intrinsic biological activities^23,24^. Recently, we demonstrated that it is possible to introduce a native peptide through an insect cuticle using a nanotechnological approach. We effectively introduced NDs coupled with *Neb*-colloostatin through the cuticle into the hemolymph of *T. molitor* individuals, where the peptide exhibits specific activity in vivo; i.e., the peptide induces hemocyte apoptosis and consequently significantly reduces the number of hemocytes circulating in the hemolymph and inhibits the cellular and humoral immune responses in all developmental stages of the insects^39^. In the current study, we also introduced the most active *Neb*-colloostatin analogs in combination with ND transcutaneously into the hemolymph of *T. molitor* larvae, pupae and adults, where the peptides effectively interfered with the cellular and humoral immune responses of the insects. The fact that [Ala^1^]-, [Val^1^]-, [Hyp^4^]- and [Ach^4^]-colloostatin and their conjugates with NDs were the most active analogs compared with the parent compound suggests that the introduction of amino acid residues that reduce the flexibility of the peptide backbone by restricting conformational freedom may have a significant impact on the penetration of the cuticle of an insect and on the biological activities of *Neb*-colloostatin.

## Conclusion

Taken together, our findings indicate that significant long-term decreases in the number of circulating hemocytes in the hemolymph of larvae, pupae and adult insects caused by strong hemocytotoxic analogs of *Neb*-colloostatin result in significant reductions in the cellular and humoral immune responses of *T. molitor* at all studied developmental stages. Among the tested *Neb*-colloostatin analogs, we identified four particularly active compounds, i.e., [Ala^1^]-, [Val^1^]-, [Hyp^4^]- and [Ach^4^]-colloostatin, which very effectively reduced the number of hemocytes circulating in the hemolymph and the short- and long-term cellular immune response in larvae, pupae and adult insects. The pronounced activity of these analogs is probably the result of not only their increased hemocytotoxicity but also the elevated stability of these compounds in the hemolymph of an insect. The modifications introduced to the *Neb*-colloostatin molecule, which significantly increased the activity of its analogs, did not adversely affect the transfer of these analogs in a complex with NDs through the insect cuticle into the hemolymph. This result confirms the possibility of the effective introduction of peptides through the cuticle and into the body of an insect while maintaining their specific activities. In this work, we did not investigate the effects of the tested analogs on insect oogenesis, embryogenesis and metamorphosis, but the pleiotropic activity of *Neb*-colloostatin suggests that its immunologically potent analogs may also more effectively inhibit ovarian development and oogenesis and may interfere with the embryogenesis and metamorphosis of insects. The identification of pleiotropic, hemolymph-stable and highly active analogs of *Neb*-colloostatin and effective methods for their introduction into insects could contribute to the development of environmentally friendly pest control methods.

## Data Availability

The datasets used during the current study are available from the corresponding author upon reasonable request.
