# Publisher Correction: Disruption of insect immunity using analogs of the pleiotropic insect peptide hormone *Neb*-colloostatin: a nanotech approach for pest control II

**DOI:** 10.1038/s41598-021-97924-x

**Published:** 2021-09-08

**Authors:** Patryk Nowicki, Mariola Kuczer, Grzegorz Schroeder, Elżbieta Czarniewska

**Affiliations:** 1 Department of Animal Physiology and Development, Institute of Experimental Biology, Faculty of Biology, Adam Mickiewicz University in Poznań, Uniwersytetu Poznańskiego Str. 6, 61-614 Poznań, Poland; 2 Faculty of Chemistry, University in Wrocław, F. Joliot-Curie Str. 14, 50-383 Wrocław, Poland; 3 Faculty of Chemistry, Adam Mickiewicz University in Poznań, Uniwersytetu Poznańskiego Str. 8, 61-614 Poznań, Poland; 4 Poznań, Poland

Correction to: *Scientific Reports* 10.1038/s41598-021-87878-5, published online 04 May 2021

In the original version of this Article Patryk Nowicki was incorrectly affiliated with ‘Department of Animal Physiology and Development, Institute of Experimental Biology, Faculty of Biology, Adam Mickiewicz University in Poznań, Uniwersytetu Poznańskiego Str. 6, 61-614, Poznań, Poland’, ‘Faculty of Chemistry, University in Wrocław, F. Joliot-Curie Str. 14, 50-383, Wrocław, Poland’, and ‘Faculty of Chemistry, Adam Mickiewicz University in Poznań, Uniwersytetu Poznańskiego Str. 8, 61-614, Poznań, Poland’. The correct affiliation is listed below.

Poznań, Poland

The original Article has been corrected.

